# Assessing the Benefit of Two Detection Approaches in Screening COVID-19 Using SARS-CoV-2 Antigen Rapid Diagnostic Tests among Intercity Public Bus Travelers in Cameroon

**DOI:** 10.4269/ajtmh.23-0778

**Published:** 2024-09-10

**Authors:** Jerome Ateudjieu, Ketina Hirma Tchio-Nighie, Anne Hoppe, Etienne Guenou, Imelda Sonia Nzinnou Mbiaketcha, Landry Beyala Bita’a, Claudine Sen Henriette Ngomtcho, Anne Cecile Bissek

**Affiliations:** ^1^ Department of Health Research, Meilleur Accès aux Soins de Santé, Yaoundé, Cameroon;; ^2^ Department of Public Health, Faculty of Medicine and Pharmaceutical Sciences, University of Dschang, Dschang, Cameroon;; ^3^ Division of Health Operations Research, Cameroon Ministry of Public Health, Yaounde, Cameroon;; ^4^ FIND, Geneva, Switzerland;; ^5^ Elizabeth Glaser Pediatric AIDS Foundation, Geneva, Switzerland;; ^6^ National Laboratory of Public Health, Yaounde, Cameroon

## Abstract

The movement of people contributes to the spread of COVID-19 between communities. Hence, we evaluated the feasibility, acceptability, and impact of offering intercity bus travelers testing prior to their departure. We conducted baseline and endline surveys to map COVID-19 prevention practices in travel agencies in western Cameroon. As interventions, buses were randomly assigned to three study arms: 1) offering systematic COVID-19 rapid diagnostic testing (RDT) to all passengers (arm A); 2) offering testing to suspected cases (arm B); or 3) no testing (arm C). All travelers were called 7–10 days after their trip to identify potential cases. Fifty-five (90.2%) of the 61 travel agencies that were reached consented to participate in a baseline survey. Although only 27 (49.1%) of the agencies implemented at least one of the recommended COVID-19 preventive measures, 39 (70.9%) agreed to host a testing station. Six agencies were selected, and 669 buses were enrolled, including 223, 224, and 222 in arms A, B, and C, respectively. A total of 31,484 departing passengers were approached and 9,594 (30.5%) agreed to participate: 1,177 (12.3%) in arm A, 4,086 (42.6%) in arm B, and 4,331 (45.1%) in arm C. In all, 1,731 tests were performed, including 1,177 in arm A and 554 in arm B. Fourteen (0.8%) tests were positive, and two participants (14.3%) agreed to postpone their travel. Offering testing with antigen RDTs in travel agencies is feasible and acceptable. One-third of passengers consented, and testing did not delay any travels. Although this approach can detect COVID-19 cases, actions are needed to increase the proportion of positive cases postponing their travels.

## INTRODUCTION

The risk of severe acute respiratory syndrome coronavirus 2 (SARS-CoV-2) transmission increases when several people travel together in an enclosed space for an extended period.^1^ There is hence a high risk of SARS-CoV-2 transmission on a public transport bus or airline.^1^^–^^3^ Measures recommended to limit the transmission of coronavirus disease 2019 (COVID-19) during transport include 1) wearing a facial mask, 2) handwashing or disinfection, 3) social or physical distancing (when possible), 4) vaccination, and 5) isolation of confirmed and contact COVID-19 cases.^4^^–^^6^ Vaccination is reported to significantly reduce COVID-19 contamination in general as well as during travel, but it does not totally eliminate transmission risk and has a variable effect on transmission depending on vaccination time, administered vaccine, and virus variants.^4^ The benefits of the application of individual preventive measures are also limited by low compliance in a large proportion of the population.^7^ The testing and quarantine of confirmed COVID-19 cases with contact tracing contribute to reducing population exposure to sources of contamination.^8^^,^^9^ The limitations of travel restriction, vaccination, testing, and application of individual preventive measures underline the need to combine these measures to minimize the spread of the disease among travelers.^4^

In Cameroon, as in many other developing countries, the majority of the population uses public buses to move between districts and cities. The West region of Cameroon is directly connected by roads to the two largest cities in Cameroon, which have international airports. A fairly large number of people travel between these cities on a daily basis by intercity bus. Intercity bus travelers are hence a potential source for spreading SARS-CoV-2 between cities and communities across the country.

We aimed to assess whether pre-departure voluntary testing of travelers using antigen rapid diagnostic tests (Ag-RDTs) is feasible and acceptable and could limit viral transmission. Antigen rapid diagnostic tests are the most affordable diagnostics available for testing for SARS-CoV-2 and can be performed in less than 30 minutes in community settings.^10^^–^^12^

## MATERIALS AND METHODS

### Study design.

The study consisted of two components: 1) a baseline survey and endline quantitative survey to map COVID-19 prevention practices in travel agencies of western Cameroon and 2) an intervention phase. The intervention phase was conducted from July to September 2022 (3 months) with variable periods per travel agency.

### Study location.

**Figure 1.**
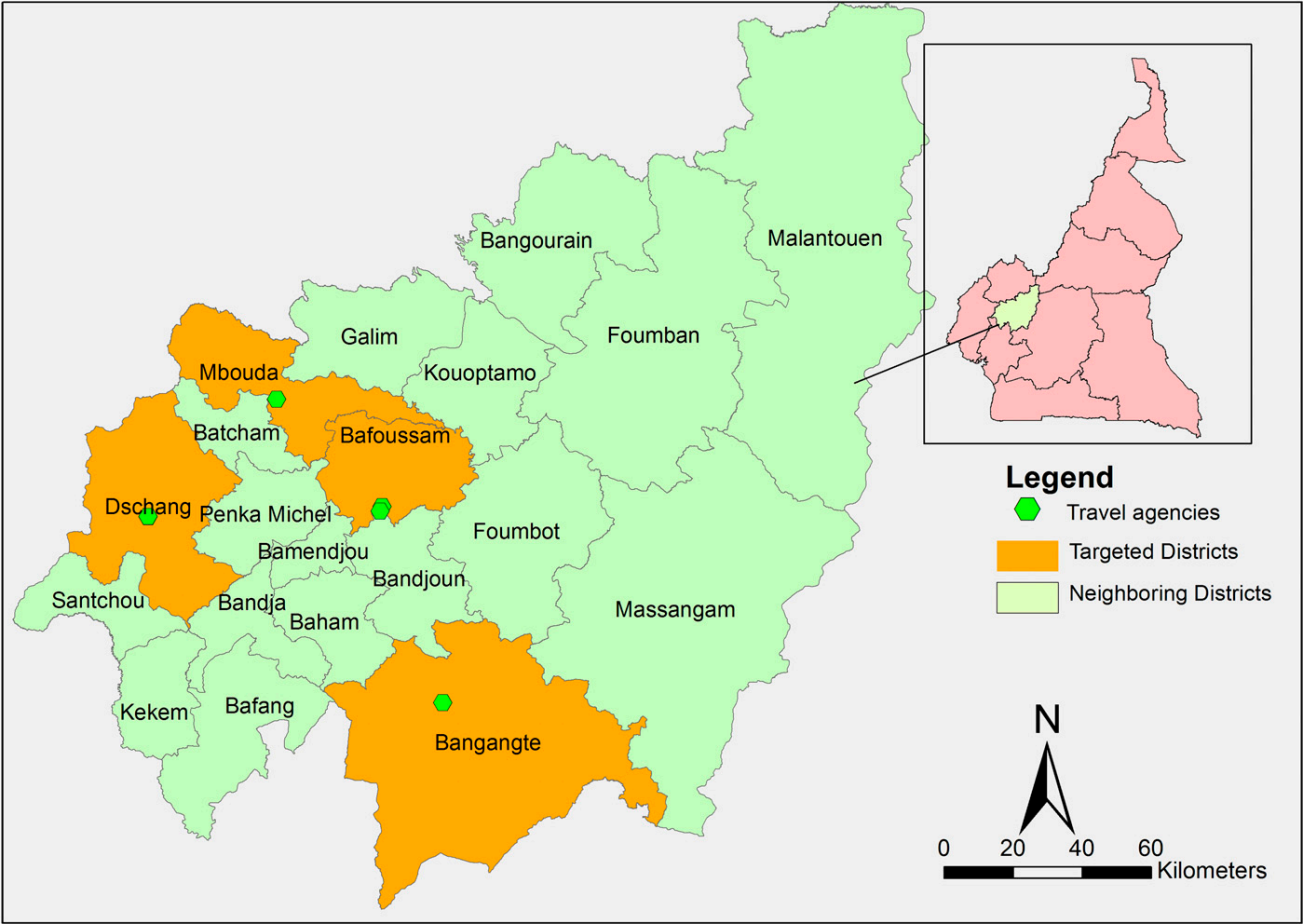
Map of the West region of Cameroon and targeted cities in the study.

The study was conducted in intercity travel agencies in four of six main cities of west Cameroon. Bafoussam, Dschang, Mbouda, and Bangangte (Figure 1) have the largest number of intercity travel agencies, and within these cities travel agencies with the greatest number of daily trips were chosen, provided they were able and willing to dedicate space for COVID-19 testing within their agency.

### Baseline and endline surveys.

Sixty-one and 68 travel agencies in western Cameroon were surveyed at baseline and after conclusion of the intervention phase, respectively, to evaluate their awareness and change in awareness with regard to the existence of COVID-19 preventive measures and implementation of these measures within their travel agencies. The baseline survey was conducted before any intervention was done in the targeted health facilities. The data were used to select the best health facilities for intervention implementation. On the other hand, the endline survey was conducted at the end of intervention implementation.

### Intervention phase.

Buses departing from selected travel agencies were randomly assigned to three study arms that involved proposing systematic COVID-19 RDTs for all passengers (arm A), selecting and proposing testing for suspected cases (arm B), or doing no testing (arm C). Testing was done per manufacturer’s instructions using the Ag-RDT test from SD Biosensor^® ^(San Diego, CA).^13^

All travelers, irrespective of group, were invited to complete an anonymous questionnaire on COVID-19. In addition, all consenting travelers were invited to provide their contact information for follow-up in 7–10 days after the travel to be remotely screened for suspected cases of COVID-19. Those suspected of having contracted SARS-CoV-2 were advised to test at their nearest facility, per national guidelines.

All travelers on selected departing buses, regardless of sex, who were 12 years or older and had registered to travel with travel tickets purchased were eligible for and invited to participate. Variables on the questionnaire included signs and symptoms recorded by the traveler during the 14 days prior to travel. These were used as the criteria to select participants for arm B. Those who did not consent to participate were not included.

### Sample size.

The minimum number of buses needed to assess the effect of the proposed intervention in each study arm was estimated at 170 buses. This was based on the following assumptions: 1) each departing bus was expected to have an average number of 32 travelers; 2) about 15% of travelers were expected to be reachable by the study team per bus; 3) about 5% of travelers in the control arm were expected to adhere to proposed COVID-19 interventions; 4) the intervention proposed in arm A or arm B would increase the proportion of people adhering to COVID-19 prevention measures to 20%, and 5) 50% of buses would spend less than 30 minutes in the travel agency and thus would not be included. This estimation was done considering a power of 90%, an error margin of 5%, and an intra-cluster correlation of 0.8.

The estimate of the sample size was done using Stata software (College Station, TX) v. 17 and was guided by the method of estimating randomized controlled cluster trials proposed by Batistatou et al.^14^

## STATISTICAL ANALYSES

All data were collected through an interface of the Open Data Kit application installed on a smartphone and validated before being transmitted to a secure password-protected database. Data cleaning happened on an ongoing basis. The proportion of travelers consenting to participate to each of the proposed interventions was estimated and compared between study arms A and B and between arms A and C using the *Z* score and *P*-value. We similarly compared the proportions for those consenting to be called 7–10 days after the trip to detect suspected cases of COVID-19. We captured the reasons of refusal to participate in any intervention and per intervention and the estimated proportion of participants who tested positive. We compared the proportion of participants with positive test results who agreed to be quarantined/postpone their travel between arms A and B. We also estimated the proportions of travelers consenting to the study interventions and the mean duration of travelers’ participation in each intervention. We also estimated and compared the incidence of suspected cases detected 7 days after traveling among the three study groups. Data were analyzed using Stata v. 17.0 IC.

## RESULTS

### Distribution and coverage of travel agencies.

Of the 81 existing travel agencies in the West region, 62 were reached and 39 of the 55 that were interviewed (70.9%) consented to host study interventions. Baseline and endline surveys were conducted among the travel agencies reached.

**Figure 2.**
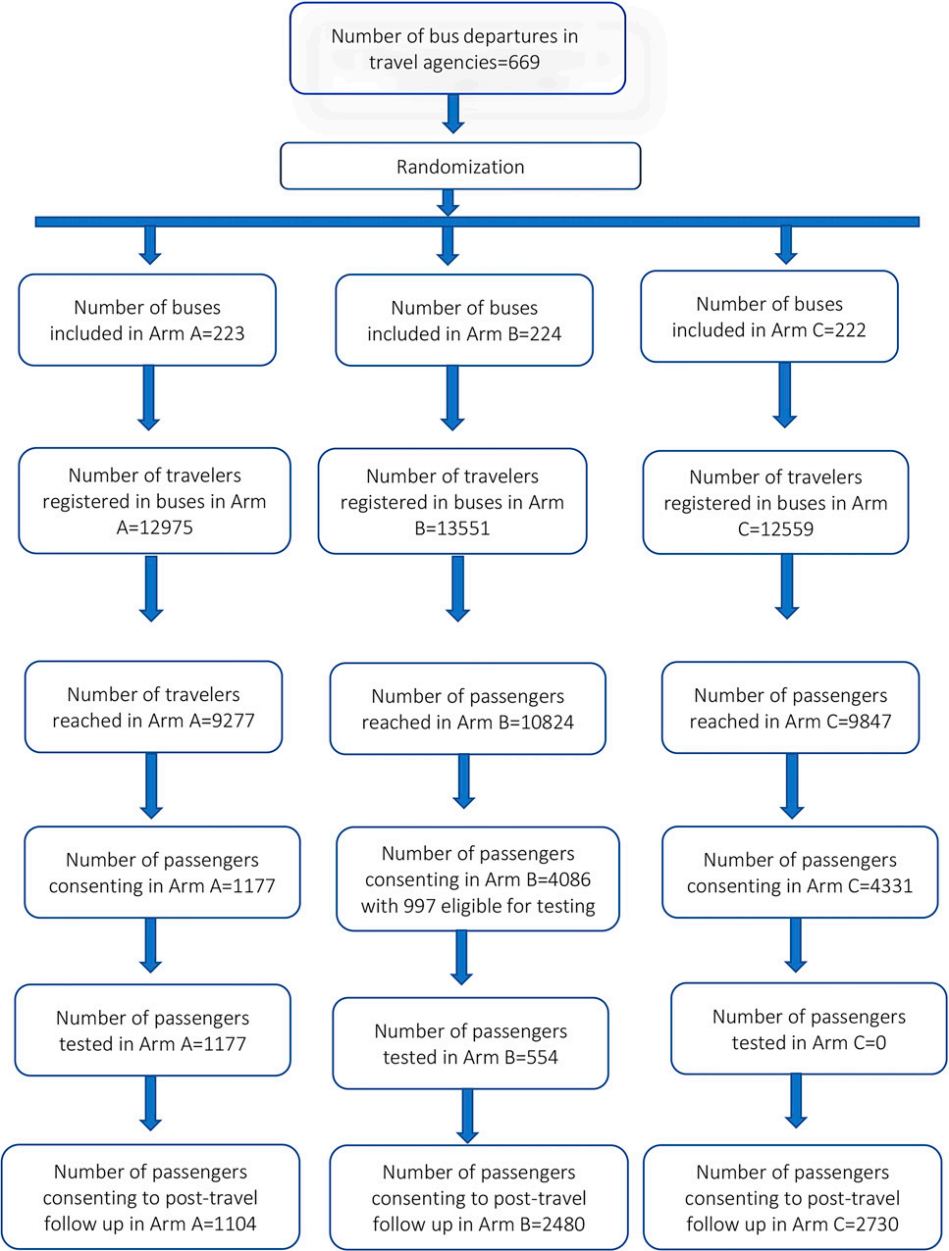
Diagram of buses and travelers included in the study.

Of those consenting to host the study interventions, six travel agencies with the highest frequency of bus departures were included in the study, including two in the city of Dschang, two in the city of Bafoussam, one in the city of Bangangte, and one in the city of Mbouda. During the study period, 669 buses were enrolled in all travel agencies (Figure 2). These included 223 in arm A, 224 in arm B, and 222 in arm C. In all travel agencies, 39,085 travelers were registered on the included buses, of whom 29,948 (76.6%) were reached by the study team.

### Baseline and endline surveys.

Of the 61 and 68 travel agencies reached during the baseline and endline surveys, 55 (90.2%) and 63 (92.7%), respectively, consented to participate. Table 1 presents the recommendations regarding COVID-19 preventive measures applied on public transport systems that respondents in travel agencies knew about as well as the measures implemented during the baseline and endline surveys. The most well-known measure at baseline was the obligation for passengers to wear a face mask (54; 98.2%) and at endline was the limitation on the number of passengers on buses (58; 92.1%), whereas the most applied measure was the setting up of a handwashing or disinfection station in travel agencies at both baseline (18; 32.7%) and endline (20; 31.7%). Only 27 (49.1%) of 55 agencies at baseline had implemented at least one of the recommended COVID-19 preventive measures.

**Table 1:** Frequencies of travel agencies knowing/implementing each of the recommended preventive measures in the baseline and endline surveys

Recommendations	Known, *n* (%)		*P*-Value	Implemented,* n* (%)		*P*-Value
	Baseline	Endline		Baseline	Endline	
Obligation of Face Mask Wearing by Travelers	54 (98.2)	56 (88.9)	0.0448	14 (25.5)	12 (19)	0.3954
Setting Up a Handwashing or Disinfection Station in Travel Agencies	53 (96.4)	56 (88.9)	0.1252	18 (32.7)	20 (31.7)	0.9076
Limitation of the Number of Passengers	47 (85.5)	58 (92.1)	0.2526	1 (1.8)	6 (9.5)	0.0384
No Overloading	46 (83.6)	52 (89.7)	0.8739	12 (21.8)	8 (12.7)	0.0375
Temperature Check at the Entry of the Agency	31 (56.4)	37 (58.7)	0.8009	0 (0.0)	6 (9.5)	–
Bus Disinfection	0 (0.0)	1 (1.6)	–	0 (0.0)	1 (1.6)	–

### Timing as a parameter of RDT testing in travel agencies.

The mean duration from the beginning of booking a bus to departure in the travel agencies was 3.04 hours (SD ±2.41 hours). For systematic testing, the mean duration of administration of the intervention was 20.54 minutes (SD ±9.64 minutes).

### Distribution of reasons for nonconsent to the study and study interventions.

**Figure 3.**
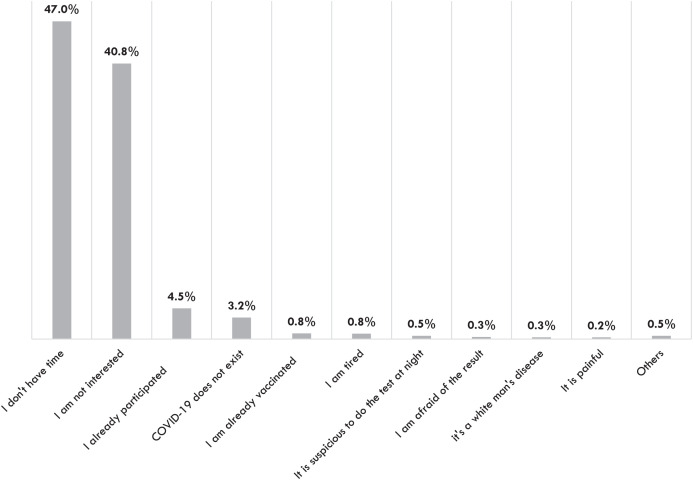
Reasons given by travelers for declining participation in the study.

**Figure 4.**
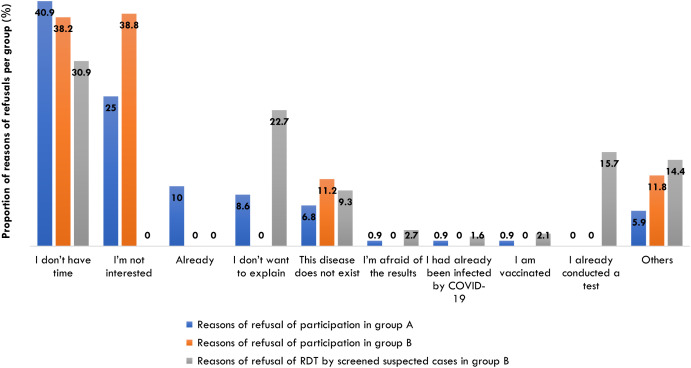
Reasons given by travelers for declining study participation and testing (arms A and B). RDT = rapid diagnostic test.

A total of 29,948 travelers were invited to participate in the study and 9,594 (32.04%) of them consented. The most common reasons given by travelers for declining participation in the study (Figure 3) and for declining any of the interventions offered as part of arms A and B (Figure 4) were lack of time and lack of interest.

### Cascade of exposure of travelers to elements of proposed interventions.

**Figure 5.**
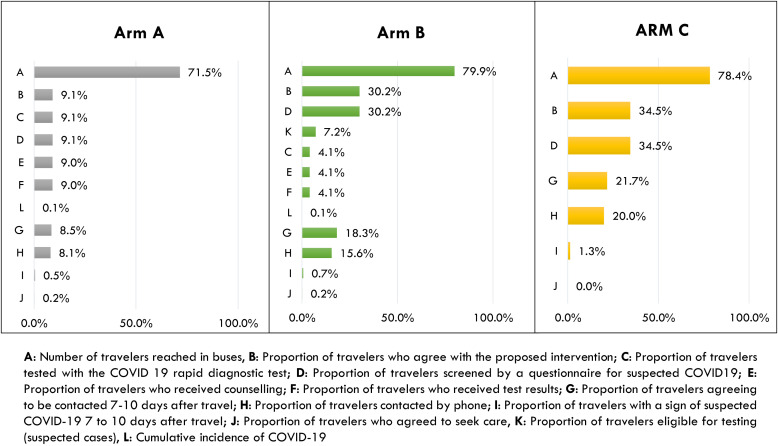
Cascade of exposure of travelers to elements of proposed interventions in study arms.

Figure 5 presents the cascade of interventions administered in the study arms. In arm A, 71.5% of travelers were reached and the intervention administered allowed to obtain the results of systematic testing and test results of 9.1% of registered travelers. In arm B, 79.9% of registered travelers were reached, and 30.2% consented to be screened to detect suspected cases. 78.4% of travelers from all the buses assigned to arm C and 34.5% consented to respond to the questionnaire.

### Travelers’ adherence to interventions proposed per study arm.

Table 2 presents estimates and comparisons between study arms of travelers’ adherence to the proposed interventions. It should be noted that travelers were less likely to consent to participate when they were offered systematic testing than when they were first screened before testing or when only their contacts were sought. A greater number of travelers were reached by phone calls in group A than in the other study groups despite approximately the same proportion of travelers having consented to phone interviews between study arms. The proportion of respondents presenting with suspected COVID-19 symptoms did not vary significantly between study arms. No difference was observed in the proportion of travelers who tested positive, and overall only 2/14 (7%) of SARS-CoV-2–positive participants were willing to postpone their trip.

**Table 2:** Distribution and comparison of travelers’ outcomes from interventions proposed per study arm and frequency and comparison of travelers’ test outcomes

Parameter	Arm A	Arm B	Arm C	Comparison Arms A/C		Comparison Arms A/B	
				Value Test (*Z* or score)	*P*-Value	Value Test (*Z* or score)	*P*-Value
Distribution and Comparison of Travelers’ Outcomes from Interventions Proposed per Study Arm							
Number Reached	9,277	10,824	9,847	–	–	–	–
Travelers Consenting to Participate, *n* (%)	1,177 (12.7)	4,086 (37.5)	4,331 (44.0)	−47.8	0.000	−39.93	0.000
Consent for Post-Travel Call (7‒10 days after the trip), *n* (%)	1,104 (93.8)	2,480 (60.7)	2,730 (63.0)	20.29	0.000	21.39	0.000
Frequency of Travelers with At Least One Symptom of COVID-19 7–10 days after the Trip	64 (5.8)	92 (3.7)	167 (6.1)	−0.35	0.724	2.85	0.004

Parameter	Arm A	Arm B	Comparison Arms A/B	
			Value Test	*P*-Value
Distribution of Frequency and Comparison of Travelers’ Test Outcomes				
Number of Travelers Eligible for Testing	1,177	979	–	–
Tested for COVID-19	1,177 (100.0)	554 (56.6)	25.22*	0.000
Counseled to Receive Results of COVID-19 Test	1,171 (99.5)	550 (99.3)	0.52*	0.605
Received Results of COVID-19 Test	1171 (99.5)	550 (99.3)	0.52*	0.605
Tested Positive for COVID-19	07 (0.6)	07 (1.3)	0.16^†^	0.123
Agreed to Postpone the Trip	2 (28.6)	0 (0.0)	0.48^†^	0.300

* *Z* test.

^†^ Fisher exact test.

## DISCUSSION

The present study was conducted to assess the feasibility and benefits of two approaches in testing for SARS-CoV-2 using Ag-RDTs among travelers prior to boarding an intercity bus. All travel agencies were aware of the existence of recommended COVID-19 preventive measures for travel agencies, but only 27 (49.1%) agencies implemented at least one of the recommended measures. Travel agencies were highly supportive of the study, with 70.9% of agencies willing and able to host a COVID-19 testing station at their terminal. Only minimal time (20.54 minutes) was required to invite, test, and counsel travelers, making it feasible to test during the check-in time (mean = 3.04 hours) and prior to their bus’s departure.

The present study revealed that all travel agencies were aware of the existence of recommendations regarding COVID-19 prevention. This implies that the dissemination of the information was appropriately implemented toward the targets. However, we noted an important gap regarding the level of implementation of the measures. This indicates a low level of monitoring and supervision of the effective implementation of recommendations that were produced by decisional authorities. Overall, knowledge and practice of prevention measures by travel agencies did not change significantly between the baseline and endline surveys. This is not surprising, given that the study did not include any interventions that could help improve these aspects.

The interest of travelers in testing varied and was lowest (13%) when systematic testing was proposed. We believe that asymptomatic individuals with no known exposure thought that the risk of not being able to travel and the potential stigma outweighed the perceived benefits of testing. Despite the difference in testing rates in arms A and B, the cumulative incidence of COVID-19 in both arms did not differ and remained low during the intervention period. The proportion of people reporting symptoms consistent with COVID-19 at 7–10 days after travel was also similar in arms A and B but was slightly higher in arm C. This was not statistically significant.

Therefore, taking human and financial resources into account, it may suffice during periods of low transmission to test only those passengers suspected of having contracted COVID-19. During a wave, intensified testing strategies may need to be used to also detect asymptomatic cases (i.e., systematic testing). This may allow the identification of symptomatic and asymptomatic cases, the detection of more cases, and travel prevention for these cases to reduce propagation. This would apply to epidemic-prone diseases for which transmission is possible during travel and/or at destinations of symptomatic, asymptomatic, or incubating carriers.

Although the study showed that it is feasible to test travelers prior to their departure and identified a potential testing strategy, the evaluated approach would have most likely been ineffective in reducing transmission on public transport during the start of a wave, as the majority of SARS-CoV-2–positive individuals were unwilling to postpone their travel. However, it is possible that the perceived risks and benefits of testing and isolation would have changed during a wave. Regardless, we believe that different approaches to improve the proportion of positive cases who agree to postpone their travel must be evaluated in the context of this or any other diseases with a similar mode of transmission. These approaches could include incentive measures and campaigns that raise awareness about the risk of transmitting and contracting diseases in crowded, poorly ventilated places when simple measures are not adhered to. We recommend that the benefit of this study be re-explored during an epidemic of a pathogen with a similar mode of transmission as SARS-CoV-2 to ensure that people are screened and isolated prior to intercity travel.

Some limitations were associated with the study. The study was conducted when the incidence of COVID-19 in the general population was low, and this not only affected human behavior but also compromised the ability to fully establish the benefits from the proposed interventions in each of the study arms. This was compounded by the fact that a large proportion of people were not approached.
